# The Efficacy, Safety, and Satisfaction Associated with Switching from Brinzolamide or Brimonidine to Brinzolamide/Brimonidine in Open-Angle Glaucoma Patients

**DOI:** 10.3390/jpm12122057

**Published:** 2022-12-13

**Authors:** Hiromitsu Onoe, Kazuyuki Hirooka, Mikio Nagayama, Hideki Mochizuki, Atsushi Hirota, Katsuyoshi Suzuki, Takeshi Sagara, Yoshiaki Kiuchi

**Affiliations:** 1 Department of Ophthalmology and Visual Science, Hiroshima University, Hiroshima 734-8551, Japan; 2 Nagayama Eye Clinic, Okayama 714-0086, Japan; 3 Kusatsu Eye Clinic, Hiroshima 733-0861, Japan; 4 Hirota Eye Clinic, Yamaguchi 745-0017, Japan; 5 Suzuki Eye Clinic, Yamaguchi 755-0155, Japan; 6 Sagara Eye Clinic, Yamaguchi 758-0021, Japan

**Keywords:** glaucoma, brinzolamide, brimonidine, satisfaction

## Abstract

We evaluated switching from brinzolamide 1% or brimonidine 0.1% to a fixed-combination of brinzolamide 1% and brimonidine 0.1%, and then determined the efficacy, safety, and satisfaction associated with these changes in glaucoma patients. This prospective, nonrandomized study evaluated a total of 31 enrolled glaucoma patients who underwent treatment with at least brinzolamide 1% or brimonidine 0.1%. Patients were administered a brinzolamide/brimonidine fixed-combination ophthalmic suspension (BBFC) after being switched from their original brinzolamide 1% or brimonidine 0.1% therapy. All other intraocular pressure (IOP)-lowering medications currently being used were continued. IOP, superficial punctate keratopathy (SPK), and conjunctival hyperemia data obtained at baseline and then at 4 and 12 weeks were evaluated. To assess the changes in treatment satisfaction, this study utilized the Treatment Satisfaction Questionnaire for Medication-9 (TSQM-9). There was a significant decrease in the mean baseline IOP from 15.7 ± 4.9 mmHg to 13.6 ± 4.4 (*p* = 0.001) and 13.5 ± 3.9 mmHg (*p* = 0.002) at 4 and 12 weeks, respectively. Evaluation of the incidence of conjunctival hyperemia or SPK score showed there were no significant changes noted at any time point. The TSQM-9 score demonstrated there was a significant increase for effectiveness after switching from brinzolamide 1% or brimonidine 0.1% to BBFC. After switching from brinzolamide 1% or brimonidine 0.1% to BBFC, there was a significant decrease in the IOP. Patients were aware of the effectiveness of switching from brinzolamide 1% or brimonidine 0.1% to BBFC.

## 1. Introduction

The second leading cause of irreversible vision loss in the world is glaucoma [1]. Intraocular pressure (IOP) elevation is the most important risk factor for glaucoma. Thus, the lowering of the IOP is considered to be the most acceptable intervention for preventing further loss of vision and for controlling glaucoma. The use of eye drops in a randomized clinical study was found to significantly lower the IOP in addition to helping to slow the disease progression [2]. The use of prostaglandin analogs has been shown to lead to the highest IOP reduction followed in descending order by β-blockers, Rho kinase inhibitors, α_2_-adrenergic agonists, and topical carbonic anhydrase inhibitors [3]. As it has also been shown that monotherapy leads to insufficient outcomes, the successful management of glaucoma requires the use of a combination therapy with multiple IOP-lowering medications [4]. However, increased numbers of medications can lead to issues with adherence, and thus, Djafari et al. [5] previously reported that the use of fewer medications led to a significantly better outcome. Therefore, regarding glaucoma treatment outcomes, adherence is one of the most important factors that is significantly associated with successful glaucoma treatment outcomes [6]. These findings suggest that fixed-combination therapies may result in better outcomes compared to treatments that administer the medications separately.

At the present time, the only fixed-combination glaucoma therapy that does not contain a β-blocker is the brinzolamide/brimonidine fixed-combination ophthalmic suspension (BBFC; Senju Pharmaceutical Co. Ltd., Osaka, Japan). In Japan, the twice-daily administration of BBFC was approved in 2019. However, if insufficient outcomes are obtained in patients treated with brinzolamide 1% or brimonidine 0.1%, the use of BBFC, rather than brinzolamide 1% plus brimonidine 0.1%, might be a better option, as this treatment uses the same eye drop bottle and the same instillation number of drops per day. The administration of BBFC as a twice-daily versus a monotherapy treatment resulted in a significantly greater IOP-lowering efficacy, compared to either of the individual components after 6 months of treatment in a previous multinational, randomized, double-masked clinical trial [7]. We recently reported that sustained IOP values were observed throughout the 12-week evaluation period after switching from brinzolamide 1% and brimonidine 0.1% to BBFC [8].

In the current study, we investigated the efficacy, safety, and satisfaction in patients who were switched from brinzolamide 1% or brimonidine 0.1% to BBFC.

## 2. Materials and Methods

From October 2020 to April 2022, this clinical trial evaluated patients at 6 investigational sites. These sites included Hiroshima University Hospital (Hiroshima, Japan), Kusatsu Eye Clinic (Hiroshima, Japan), Hirota Eye Clinic (Yamaguchi, Japan), Nagayama Eye Clinic (Okayama, Japan), Suzuki Eye Clinic (Yamaguchi, Japan), and Sagara Eye Clinic (Yamaguchi, Japan). The Institutional Review Board of the Hiroshima University approved the study protocol (E-2184). In accordance with the principles outlined in the Declaration of Helsinki, all subjects provided their informed written consent to participate in this study.

All patients who took part in this study underwent examinations that included visual acuity, refraction, visual field, slit-lamp examination, and gonioscopy. The eligibility criteria used in the study included having an age ≥ 20 years, glaucomatous optic disc changes, and corresponding glaucomatous visual field defects. Glaucomatous optic disc changes were defined as having a vertical cup-disc asymmetry between the fellow eyes that was ≥0.2, a cup-to-disc ratio that was ≥0.6, and an eye that exhibited neuroretinal rim narrowing, notches, localized pallor, or retinal nerve fiber layer (RNFL) defects. Glaucomatous visual field defects were defined according to the Anderson–Patella criteria [9]. Furthermore, to be enrolled in the study, patients had to be treated with at least brinzolamide 1% (Alcon Laboratories, Inc., Fort Worth, TX, USA) or brimonidine 0.1% (Senju Pharmaceutical Co. Ltd., Osaka, Japan). Patients who had active ocular diseases in either eye except for glaucoma, had undergone ocular surgery or laser treatment within 1 year previously, were on a regimen during the study period for systemic or local administration of steroid, or had corneal disease in either eye that potentially posed a problem for accurate IOP measurements, were all excluded from the study analysis. When the inclusion criteria were met by both eyes of the patient, the eye with the higher IOP at baseline was used for the analysis. If the IOP measurements were the same in both eyes, the right eye was used in the analysis.

**Figure 1:**
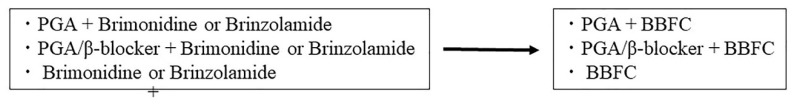
IOP-lowering medications.

A total of 3 patient visits were scheduled over 12 weeks (day 0 and weeks 4 and 12) during the study period. Patients were deemed eligible for enrollment if at day 0 (baseline) they were using brinzolamide 1% (twice daily) or brimonidine 0.1% (twice daily), after which they were switched to BBFC (twice daily). All other IOP-lowering medications used remained the same (Figure 1). The administration time of BBFC was set to approximately the same time as that for the administration of brinzolamide 1% or brimonidine 0.1%. An insufficient IOP was the main reason why patients were switched to BBFC.

IOP measurements, best-corrected visual acuity, and biomicroscopic examinations were performed in all patients at each visit. The same procedure was performed at all centers using a Goldmann applanation tonometer, with measurements of the IOP performed at the same time as the administration of the brinzolamide 1% or brimonidine 0.1%. The primary IOP change observed at 12 weeks from the baseline was defined as the primary efficacy outcome. Before implementing any changes, average baseline IOPs were determined twice prior to switching the patients to BBFC.

For biomicroscopy evaluations, slit-lamp examinations were performed in all patients. A 4-point hyperemia grading scale that utilized four different photographs to determine the hyperemia matching was utilized in order to assess the conjunctival hyperemia. The grading on this scale was set as: 0 = no hyperemia, 1 = slight hyperemia, 2 = moderate hyperemia, and 3 = severe hyperemia. During the slit-lamp examinations, corneal epithelial disorder recordings used an A (area) and D (density) grading scale [10].

In order to assess the change in treatment satisfaction, patients were evaluated using the Treatment Satisfaction Questionnaire for Medication-9 (TSQM-9) [11], which utilizes a 5- or 7-point scale in order to determine the assessment. Increases in the scores were indicative of an increase in the patient’s treatment satisfaction. Results were further grouped in order to evaluate the effectiveness, convenience, and global satisfaction scores. Total scores ranged from 0 to 100 in these three evaluated areas. A higher score was indicative of greater satisfaction. Questionnaires were completed by all patients both at the baseline, and again at 12 weeks.

The Anderson–Daring test was used to assess the variance equality for the continuous variables. Differences between the baseline and follow-up visits for the obtained results were subsequently assessed utilizing either a Student’s *t*-test or a Wilcoxon signed-rank test. TSQM-9 scores are presented as the median and interquartile ranges. All statistical analyses were conducted using JMP software version 16 (SAS Inc., Cary, NC, USA). A *p* value less than 0.05 was considered statistically significant. The data are presented as the mean ± standard deviation.

## 3. Results

Of the 34 patients who were initially enrolled, the side effects noted included conjunctivitis in 1 patient, blurred vision in 1 patient, and blepharitis in 1 patient within 4 weeks after switching BBFC. Therefore, a total of 3 patients were removed from the overall study analysis. As a result, there were 31 total patients (31 eyes) who completed the 12-week follow-up. Table 1 presents the baseline patient demographic data. The mean age was 69.9 ± 10.1 (range, 46 to 86) years, with a total of 18 eyes switched from brinzolamide 1% to BBFC. In addition, there were 13 eyes that were switched from brimonidine 0.1% to BBFC. The mean durations of the treatments with brinzolamide 1% or brimonidine 0.1% were 21.8 ± 30.1 (range, 1 to 123) months or 28.0 ± 18.6 (range, 1 to 67) months, respectively. There were 28 eyes that had primary open-angle glaucoma and 3 eyes that had exfoliation glaucoma. The mean deviation of the Humphrey visual field test program (30-2 SITA standard) was −8.3 ± 6.2 (range, −29.76 to −0.73) dB.

**Figure 2:**
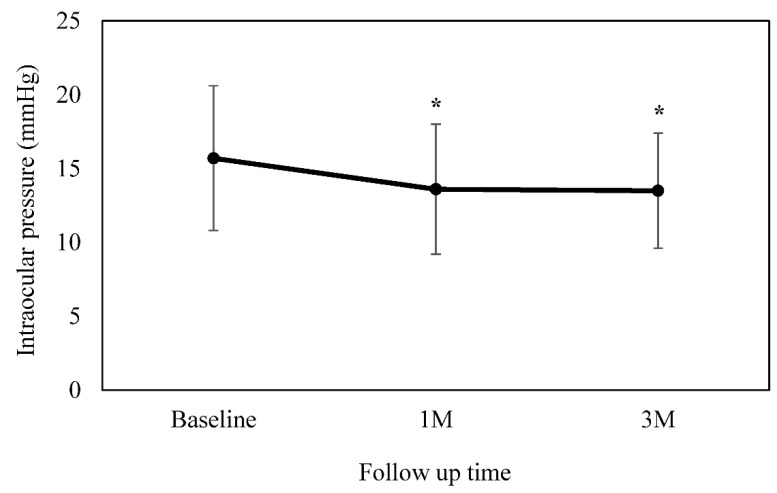
Mean intraocular pressure at baseline and at 4 and 12 weeks. There was a significant decrease observed in the mean IOP as compared to the baseline. This is figure legend of Figure 2. * *p* < 0.05.

The IOP at baseline was 15.7 ± 4.9 mmHg. The IOP at 4 weeks was 13.6 ± 4.4 mmHg (*p* = 0.001), while at 12 weeks it was 13.5 ± 3.9 mmHg (*p* = 0.002) (Figure 2). Analysis of the data between the baseline and each of the follow-up visits showed there were significant differences. The mean reduction in IOP was 2.4 ± 2.6 mmHg after 4 weeks, and 2.3 ± 4.2 mmHg after 12 weeks. The IOP at baseline when switching from brinzolamide 1% to BBFC was 17.0 ± 5.6 mmHg, which then significantly decreased to 14.5 ± 4.9 mmHg (*p* = 0.007) and 14.5 ± 4.1 mmHg (*p* = 0.03) at 4 and 12 weeks, respectively. In contrast, the IOP at baseline when switching from brimonidine 0.1% to BBFC was 14.0 ± 2.9 mmHg, which then significantly decreased to 12.4 ± 3.1 mmHg (*p* = 0.008) and 12.2 ± 3.4 mmHg (*p* = 0.02) at 4 and 12 weeks, respectively.

Data assessed when using the AD grading scale along with the conjunctival hyperemia scores are presented in Table 2. This table also shows the degree of the SPK. No significant differences were observed for any of the evaluated time points. There were 20 patients that had a total SPK score of 0, 8 patients that had a total SPK score of 2, and 3 patients that had a total SPK score of 3 at baseline. Although 6 eyes exhibited an increase in SPK scores, 6 eyes exhibited a decrease in SPK scores after 12 weeks.

TSQM-9 scores obtained at baseline and at 12 weeks are presented in Table 3. The TSQM-9 was not completed by 3 patients, who discontinued the study treatment before 12 weeks. As a result, data were only available for 31 patients for the analysis. Convenience and global satisfaction scores were also obtained at baseline and at 12 weeks. TSQM-9 baseline score comparisons indicated that patients were satisfied with the effectiveness.

## 4. Discussion

Although the conjunctival hyperemia and degree of SPK were sustained throughout the 12-week observation period, there was a significant decrease in the IOP values after switching to BBFC from brinzolamide 1% or brimonidine 0.1%. In addition, patients indicated that they were satisfied with the effectiveness of the therapy.

If the IOP cannot be successfully lowered to the exact target pressure, but the therapy appears to be tolerated and effective, then the use of an additional drug of a different class needs to be taken into consideration. However, when using multiple topical treatments, there is a chance that this could reduce adherence and potentially increase the exposure to the preservatives contained within the administered agents. Therefore, instead of using two separate instillations of these agents, the preferred treatment is to use a fixed-combination therapy. Moreover, it has been shown in a previous study that after to switching BBFC from brinzolamide or brimonidine, there was a significant decrease in the IOP [12]. Other studies have also reported that BBFC has an IOP-lowering efficacy that is similar to that found for brinzolamide plus brimonidine [8,12,13]. Indeed, after switching from brinzolamide 1% or brimonidine 0.1% to BBFC in the present study, we also found that there was a significant decrease in the IOP.

There are several advantages associated when using fixed-combination medication compared to the concomitant dosing of two separate medications. For example, when using two separate medication dosages, this can potentially cause a washout of the first medication, thereby leading to an insufficient therapeutic effect [14]. In order to overcome these issues when using 2 separate medications, an interval of at least 5 min should be utilized between the 2 eye drops. Use of this administration technique will help prevent any potential washout of the first medication due to the instillation of the second agent [15]. With regard to adherence, Djafari et al. [5] have previously reported that there was a significant association between fewer medications and patient adherence. Furthermore, as previously mentioned, when using multiple eye drops this can cause an increased exposure to the preservatives used in the ophthalmic solutions. It has been reported that there is an association between the use of multiple eye drops containing preservatives and increased ocular surface diseases [8].

The use of the TSQM-9 to assess patients’ satisfaction with treatments has been shown to be reliable, with this questionnaire having been utilized to evaluate treatment satisfaction assessments for various diseases [11,16]. In our previous study, we also evaluated patients’ satisfaction after switching to BBFC from brinzolamide and brimonidine by using the TSQM-9 [8]. Our analysis showed that there was an increase in both the convenience and satisfaction scores after switching to BBFC from brinzolamide and brimonidine [8]. Moreover, our current study also found that there was a significant decrease in the IOP after switching from brinzolamide or brimonidine, even though the same eye drop bottles were used. The current study results also demonstrated that there was an increase in the effectiveness from the baseline 50 to 72. However, as has been previously pointed out, it should be noted that there are many factors that can influence glaucoma treatment adherence [17]. Lack of motivation has been shown to be one of the obstacles to adherence by patients [18]. In our current study, however, after patients switched to BBFC, they began to notice and understand the effectiveness of this treatment due to associated decreases in the IOP. These results suggest that there could be an improvement in patient motivation if patients are aware of the decrease that occurs in the IOP.

In our current study, there were several limitations. First, there was only a small sample size analyzed in this study. Thus, in order to obtain more rigorous, comparative, and definitive evidence and data, multi-center and large-scale trials will need to be undertaken in the future. Another limitation was that the follow-up period in our current study might not have been long enough. Results closer to what is actually observed in routine practice could potentially be obtained by increasing the experimental follow-up period. At the present time, the mean follow-up period after switching to BBFC was 17.5 ± 8.7 (range, 4 to 25) months. Of the 31 patients who were analyzed in this study, the side effects included conjunctivitis in 2 patients, at 6 and 11 weeks after switching to BBFC; blepharitis in 3 patients, at 4, 6, and 24 months after switching; and episcleritis in 1 patient after the 12 week-evaluation period. There were 2 patients that had an insufficient IOP and thus, subsequently underwent glaucoma surgery at 5 and 14 months after switching to BBFC. Third, when switching from brinzolamide or brimonidine to BBFC, there are presently no defined criteria with regard to how this needs to be implemented. All of these changes were strictly made based on the decision of the attending physicians. The progression of glaucomatous visual field damage due to an insufficient IOP was the primary reason why patients were switched to BBFC.

In conclusion, after switching to BBFC from brinzolamide 1% or brimonidine 0.1%, the current results showed that there was a significant decrease in the IOP. Furthermore, patients were aware of the effectiveness of this change in their therapy after switching to BBFC from brinzolamide 1% or brimonidine 0.1%.

## Figures and Tables

**Table 1:** Clinical characteristics of study patients.

Age (years)	69.9 ± 10.1
Gender (M/F)	11/20
Baseline IOP (mmHg)	15.7 ± 4.8
Mean deviation (dB)	−8.3 ± 6.2
Type of glaucoma	
POAG	28
Exfoliation glaucoma	3
Brinzolamide/brimonidine	18/13
IOP-lowering medications	
(except brimonidine and brinzolamide)	
PGA	15
PGA/β-blocker	9
None	7

M; male, F; female, POAG; primary open-angle glaucoma; IOP; intraocular pressure, PGA; prostaglandin analogue.

**Table 2:** SPK and hyperemia scores.

	SPK Score	*p* Value *	Hyperemia Score	*p* Value *
Baseline	0.66 ± 1.00		0.28 ± 0.46	
1M	0.89 ± 1.17	0.24	0.32 ± 0.48	0.33
3M	0.72 ± 1.16	0.54	0.33 ± 0.61	0.71

SPK; superficial punctate keratopathy; * Calculated using Wilcoxon signed-rank test.

**Table 3:** TSQM-9 scores.

	Baseline (IQR)	3M (IQR)	*p* Value *
Effectiveness	50 (50–67)	67 (50–70)	0.04
Convenience	67 (63–78)	67 (67–83)	0.47
Global satisfaction	57 (52–71)	60 (52–71)	0.61

IQR; interquartile range, *; Calculated using Wilcoxon signed-rank test.

## Data Availability

The data analyzed in this study are available from the corresponding author on reasonable request.
